# Feasibility and preliminary signal of cerebellar intermittent theta burst stimulation for static balance in cerebellar ataxia: a pilot study

**DOI:** 10.3389/fneur.2026.1826602

**Published:** 2026-07-15

**Authors:** Sumin Lee, Eunhee Park, Yongjeon Cheong, Jihyeong Ro, Jungeun Kim, Ho-Won Lee, Minyoung Jung

**Affiliations:** 1 Cognitive Science Research Group, Korea Brain Research Institute, Daegu, Republic of Korea; 2 Department of Artificial Intelligence, Kyungpook National University, Daegu, Republic of Korea; 3 Department of Rehabilitation Medicine, School of Medicine, Kyungpook National University, Daegu, Republic of Korea; 4 Department of Biomedical Science, Kyungpook National University, Daegu, Republic of Korea; 5 Department of Neurology, Kyungpook National University Chilgok Hospital, Daegu, Republic of Korea; 6 Department of Neurology, School of Medicine, Kyungpook National University, Daegu, Republic of Korea; 7 Brain Science and Engineering Institute, Kyungpook National University, Daegu, Republic of Korea

**Keywords:** balance, cerebellar ataxia, fNIRS, iTBS, motor–cognitive interaction

## Abstract

This study explored the short-term effects of 50 Hz cerebellar intermittent theta burst stimulation (iTBS) on static balance and prefrontal cortical activation in patients with cerebellar ataxia (CA). Ten patients with CA (4 males; mean age, 57.6 ± 9.1 years) underwent iTBS over the bilateral cerebellum for five consecutive days (8 min/session, 80% active motor threshold); static balance was assessed using a Balance Trainer 4 (BT4) under eyes-open (EO) and eyes-closed (EC) conditions at baseline (V0), immediately post-intervention (V1), and 6 weeks post-treatment (V2). Clinical scales (K-SARA, K-MBI, SF-36-K) and functional near-infrared spectroscopy (fNIRS) during a dual-task toe-tapping paradigm (motor + mental subtraction) were also administered to evaluate motor and cognitive responses. Significant, albeit transient, improvements in postural balance were observed at V1 relative to V0: under EO conditions, anterior–posterior sway (ΔY) and total displacement (Δd) decreased significantly (*p* < 0.05), and under EC conditions, medial–lateral sway (ΔX) also improved (*p* < 0.05); however, these effects were not sustained at V2. Regarding safety, dizziness was the most frequently reported adverse event, occurring in 5 of 10 participants (50%); all events were mild–moderate, transient, and resolved without treatment discontinuation. In conclusion, 50 Hz cerebellar iTBS was well tolerated and transiently enhanced postural stability in CA patients, and combined fNIRS monitoring demonstrated cortical engagement during cognitive-motor integration, supporting fNIRS as a real-time brain imaging biomarker for exploring the neurophysiological mechanisms underlying iTBS-induced modulation of the cerebello-fronto-cortical loop.

## Introduction

Cerebellar ataxia (CA) is a neurodegenerative disorder resulting from cerebellar dysfunction, primarily characterized by severe balance impairment and gait instability (1–3). The cerebellum plays a crucial role not only in motor coordination but also in maintaining postural balance through the integration of visual, proprioceptive, and vestibular feedback (4–6). Beyond its sensorimotor functions, the cerebellum is increasingly recognized as a key node within distributed networks supporting cognitive, attentional, and executive processing (6, 7). Through reciprocal cerebello-cerebral loops linking the cerebellum with prefrontal and parietal association cortices, the cerebellum contributes to higher-order functions such as working memory, attentional control, and motor–cognitive integration (6, 7). These functions are particularly engaged during dual-task performance, in which concurrent motor and cognitive demands require coordinated recruitment of prefrontal executive resources together with cerebellar timing and prediction (8, 9). Consequently, cerebellar dysfunction in CA may compromise not only postural control but also the cortical resources recruited when motor and cognitive demands are processed concurrently, providing a clear rationale for probing the cerebello-fronto-cortical loop with a dual-task paradigm (7, 9).

Repetitive transcranial magnetic stimulation (rTMS), a non-invasive neuromodulatory technique, has shown potential to modulate abnormal cerebello-thalamo-cortical connectivity and improve symptoms in various neurological disorders, including Parkinson’s disease and cerebellar syndromes (10–13). High-frequency rTMS exerts excitatory effects on neural networks and has been shown to enhance motor performance when applied to cerebellar regions (14–16). While most studies have utilized low- to moderate-frequency stimulation (1–20 Hz) (11, 17, 18), emerging evidence suggests that 50 Hz stimulation may induce stronger and faster modulation of cerebello-thalamo-cortical connectivity by reducing cerebellar brain inhibition (CBI) and facilitating cortical excitability through Purkinje cell disinhibition (19, 20). This study explored the safety, tolerability, and potential therapeutic effects of 50 Hz cerebellar iTBS on postural balance in patients with CA.

To directly visualize and validate the cortical mechanisms by which cerebellar iTBS reshapes motor–cognitive networks, we incorporated functional near-infrared spectroscopy (fNIRS) during a toe-tapping paradigm administered under two conditions: a single-task motor condition (toe-tapping alone) and a dual-task condition combining toe-tapping with concurrent mental subtraction (9, 21, 22). The single-task condition was retained as an isolated motor reference, allowing us to characterize pure motor-related prefrontal activity, whereas the dual-task condition was added to load the motor task with an executive component and thereby probe motor–cognitive integration (8, 9). The deliberate contrast between the two conditions (dual-task minus single-task) isolates the cortical cost specifically attributable to the added cognitive demand and provides a more sensitive probe of iTBS-induced modulation of the cerebello-fronto-cortical loop than either condition alone. This design choice reflects the observation that simple self-paced toe-tapping elicits limited and variable prefrontal hemodynamic responses, whereas the dual-task contrast reliably engages prefrontal executive resources thought to be supported by cerebello-cerebral loops and disrupted in CA (8, 22, 23). Unlike conventional clinical rating scales, fNIRS enables continuous, non-invasive, real-time monitoring of prefrontal oxygenation at the bedside, serving as a brain imaging biomarker that directly reflects the engagement of the cerebello-fronto-cortical loop during cognitive-motor integration (21, 23).

This pilot study thus aimed to evaluate both the behavioral and cortical responses to short-term, high-frequency cerebellar iTBS. Crucially, by coupling 50 Hz cerebellar iTBS with simultaneous fNIRS brain monitoring, we sought to go beyond simply observing clinical outcomes and instead directly visualize and verify the neurophysiological mechanisms through which iTBS modulates the cerebello-fronto-cortical loop. This multimodal approach provides preliminary evidence that fNIRS-derived cortical hemodynamics may serve as a candidate brain imaging biomarker of iTBS-induced neuromodulation in cerebellar ataxia, offering a preliminary framework for exploring how cerebellar stimulation may influence cortical networks involved in postural stability. In alignment with the overarching focus of this Research Topic on brain imaging and stimulation for motor control and movement disorders, the present study represents one of the first pilot investigations to deploy fNIRS as a real-time neuroimaging tool for tracking iTBS-induced neuromodulation of the cerebello-fronto-cortical loop, supporting its potential as a candidate brain imaging biomarker for exploring non-invasive neuromodulation in cerebellar ataxia.

## Method

### Participants and design

Ten patients with clinically diagnosed CA (4 males; mean ± SD = 58 ± 9.1 years) participated in the study. Given the exploratory, proof-of-concept nature of this Phase 1 investigation and the low prevalence of cerebellar ataxia, the sample size was deliberately small and was selected to establish safety, tolerability, and feasibility rather than to provide a definitive efficacy estimate; the resulting limitations in statistical power and generalizability are explicitly discussed in the Limitations section. The cohort comprised three diagnostic subgroups: multiple system atrophy–cerebellar type (MSA-C, *n* = 5), sporadic adult-onset ataxia (SAOA, *n* = 4), and spinocerebellar ataxia type 6 (SCA6, *n* = 1; genetically confirmed). All SAOA participants were diagnosed by exclusion on the basis of (a) negative genetic testing for SCA1, 2, 3, 6, 7 and DRPLA, (b) brain MRI showing isolated cerebellar atrophy without brainstem or putaminal signal changes suggestive of MSA-C, (c) biochemical and serological screening (B12, vitamin E, thyroid function, anti-gliadin, anti-transglutaminase, and anti-GAD antibodies) with negative findings, and (d) autonomic evaluation confirming the absence of severe autonomic failure (see Supplementary Information for full details). All procedures were approved by the Kyungpook National University Chilgok Hospital IRB and the Ministry of Food and Drug Safety. Exclusion criteria included epilepsy, metal implants, and severe cognitive impairment. Detailed procedures and assessment protocols are described in the Supplementary Figure S1 for the experimental protocol.

### iTBS intervention

iTBS was delivered bilaterally to the cerebellum using a figure-of-eight coil at 80% of the active motor threshold (8 min/session × 5 days, 1,200 pulses/session). iTBS consists of bursts of three pulses at 50 Hz repeated every 200 ms and was applied in 2 s trains repeated every 10 s. Participants were positioned seated comfortably with head support. The cerebellar stimulation sites were localized 1 cm below and 4 cm lateral to the inion. The order of bilateral stimulation was left then right. Stimulation was delivered in accordance with established safety guidelines (19, 20, 24).

Participants were monitored for adverse events during and after each session using systematic self-report and examiner observation. Detailed safety and tolerability outcomes are presented in the Results section.

### Assessments

Data were collected at baseline (V0), immediately post-treatment (V1), and at a 6-week follow-up (V2). The Korean version of the Scale for the Assessment and Rating of Ataxia (K-SARA), the Modified Barthel Index (K-MBI), and the 36-Item Short Form Health Survey (SF-36-K) were used. These instruments evaluate ataxia severity, functional independence, and quality of life, respectively. Static balance was measured using a Balance Trainer 4 (BT4; HUR, Finland) under eyes-open (EO) and eyes-closed (EC) conditions. Center of pressure (COP) displacement along the medial-lateral (X) and anterior–posterior (Y) axes was analyzed, including velocity, total displacement (d), and Romberg quotient (RQ). The delta parameters (ΔX, ΔY, and Δd) represent the displacement changes calculated every 10 ms. Cognitive-motor interaction was examined using the NIRSIT Lite (OBELAB, Korea), a 15-channel functional near-infrared spectroscopy (fNIRS) device, during left/right toe-tapping tasks with and without mental subtraction. The device recorded changes in oxygenated and deoxygenated hemoglobin concentrations using wavelengths of 780 and 850 nm at a sampling rate of 8.138 Hz.

### Statistical analysis

Non-parametric Friedman tests were used to assess time-dependent changes (V0–V2), followed by Wilcoxon signed-rank *post hoc* comparisons. Statistical significance was set at *p* < 0.05. Exploratory Spearman rank correlations were used to relate the V1-to-V0 increase in R-MFG oxygenated hemoglobin during the LDT condition to the V1-to-V0 reduction in medial–lateral sway (SR_ML), computed as the V0 baseline value minus the V1 post-treatment value so that positive values denote reduced sway (improved balance). Note that ∆X in Supplementary Table S2 denotes the medial–lateral sway displacement measured at each visit, whereas the variable entered into the correlation and plotted in Supplementary Figure S3 is the V1-to-V0 reduction in this measure (SR_ML); the two should not be confused. More details of clinical assessment are described in the Supplementary Information.

## Results

The iTBS treatment was well-tolerated by all participants, and detailed demographic and clinical characteristics of the cohort are provided in Table 1 (main text presents demographic and clinical scale data, whereas Supplementary Table S1 reports the inferential statistics for prefrontal fNIRS activation; the two tables are complementary and non-overlapping). Safety and tolerability: No serious adverse events (SAEs) and no seizures were recorded. Dizziness was the most frequently reported adverse event, occurring in 5 of 10 participants (50%) at least once during the intervention period. In four cases, dizziness was mild (CTCAE Grade 1), characterized by transient lightheadedness that lasted less than 30 min following bilateral cerebellar stimulation and did not require modification of the treatment schedule. One participant experienced moderate dizziness (CTCAE Grade 2) persisting for approximately 24 h, necessitating a one-day postponement of the third stimulation session; the remaining sessions were subsequently completed without further interruption. Local scalp discomfort during stimulation was reported by two participants (Grade 1), and one participant reported mild headache lasting approximately 2 h post-session (Grade 1), which resolved spontaneously without analgesics. All symptoms resolved completely within 1 week of the final stimulation session, and no participant discontinued the study due to adverse events. The Friedman test revealed a significant effect of the intervention on static balance performance (Supplementary Table S2). In the EO condition, there were significant improvements at V1 for the maximum value of anterior–posterior sway (∆Y max: *Q* = 8.6, *p* = 0.014, *W* = 0.43; ∆Y mean: *Q* = 8.6, *p* = 0.014, *W* = 0.43) and total sway distance (∆d mean: *Q* = 8.6, *p* = 0.014, *W* = 0.43; ∆d STD: *Q* = 7.2, *p* = 0.027, *W* = 0.36). In the EC condition, significant improvements were observed in the mean and STD of medial-lateral sway (∆X mean: *Q* = 6.2, *p* = 0.045, *W* = 0.31; ∆X STD: *Q* = 7.2, *p* = 0.027, *W* = 0.36). Total sway displacement (∆d) is reported above as a primary balance outcome. Sway velocity and the Romberg quotient (RQ) were also derived from the BT4 recordings and entered into the Friedman analysis. Sway velocity showed no statistically significant change across the three time points (all *p* > 0.05), whereas the Romberg quotient showed a significant Friedman effect (*p* = 0.0004) that did not survive post-hoc Wilcoxon signed-rank comparison; neither is therefore discussed further. Additional gait outcomes, including velocity and RQ, are provided in Supplementary Tables S2, S3 and Supplementary Figure S4.

**Table 1:** Demographic and clinical characteristics of patients with cerebellar ataxia, by diagnostic subgroup.

Variable	MSA-C	SAOA	SCA	Total
*n*	5	4	1	10
Age (years)	57.8 ± 11.5	56.3 ± 7.9	62	57.6 ± 9.1
Sex (M/F)	3/2	1/3	0/1	4/6
Disease duration (years)	3.4 ± 1.9	4.3 ± 3.4	4	3.8 ± 2.4
K-SARA				
Pre	14.7 ± 7.5	14.5 ± 7.8	7.5	13.9 ± 7.1
Post	13.0 ± 6.7	12.4 ± 7.9	5.5	12.0 ± 6.8
Follow-up	12.0 ± 5.7	14.0 ± 5.9	5.5	12.2 ± 5.7
K-MBI				
Pre	53.0 ± 29.5	47.0 ± 27.0	82	53.5 ± 27.2
Post	53.0 ± 29.5	47.0 ± 27.0	82	53.5 ± 27.2
Follow-up	52.6 ± 29.0	44.0 ± 28.1	82	52.1 ± 27.7
SF-36 (total)				
Pre	34.8 ± 28.0	29.8 ± 11.9	45	33.8 ± 20.4
Post	36.4 ± 27.3	30.0 ± 12.3	41	34.3 ± 19.9
Follow-up	36.4 ± 21.0	30.0 ± 5.7	37	33.9 ± 14.8
SF-36 (physical health)				
Pre	32.2 ± 24.4	26.3 ± 8.7	48	31.4 ± 18.2
Post	35.8 ± 25.6	27.8 ± 10.3	39	32.9 ± 18.6
Follow-up	32.6 ± 16.5	25.8 ± 4.1	41	30.7 ± 12.3
SF-36 (mental health)				
Pre	33.0 ± 26.6	32.3 ± 17.7	42	33.6 ± 20.7
Post	34.8 ± 26.9	32.5 ± 14.9	41	34.5 ± 20.3
Follow-up	34.0 ± 24.5	33.0 ± 8.8	22	32.4 ± 17.5

MSA-C, Multiple System Atrophy – Cerebellar type; SAOA, Sporadic Adult-Onset Ataxia; SCA, Spinocerebellar Ataxia; K-SARA, Korean version of the Scale for the Assessment and Rating of Ataxia; K-MBI, Korean Modified Barthel Index; SF-36, 36-Item Short Form Health Survey; PH, Physical Health; MH, Mental Health.

**Figure 1:**
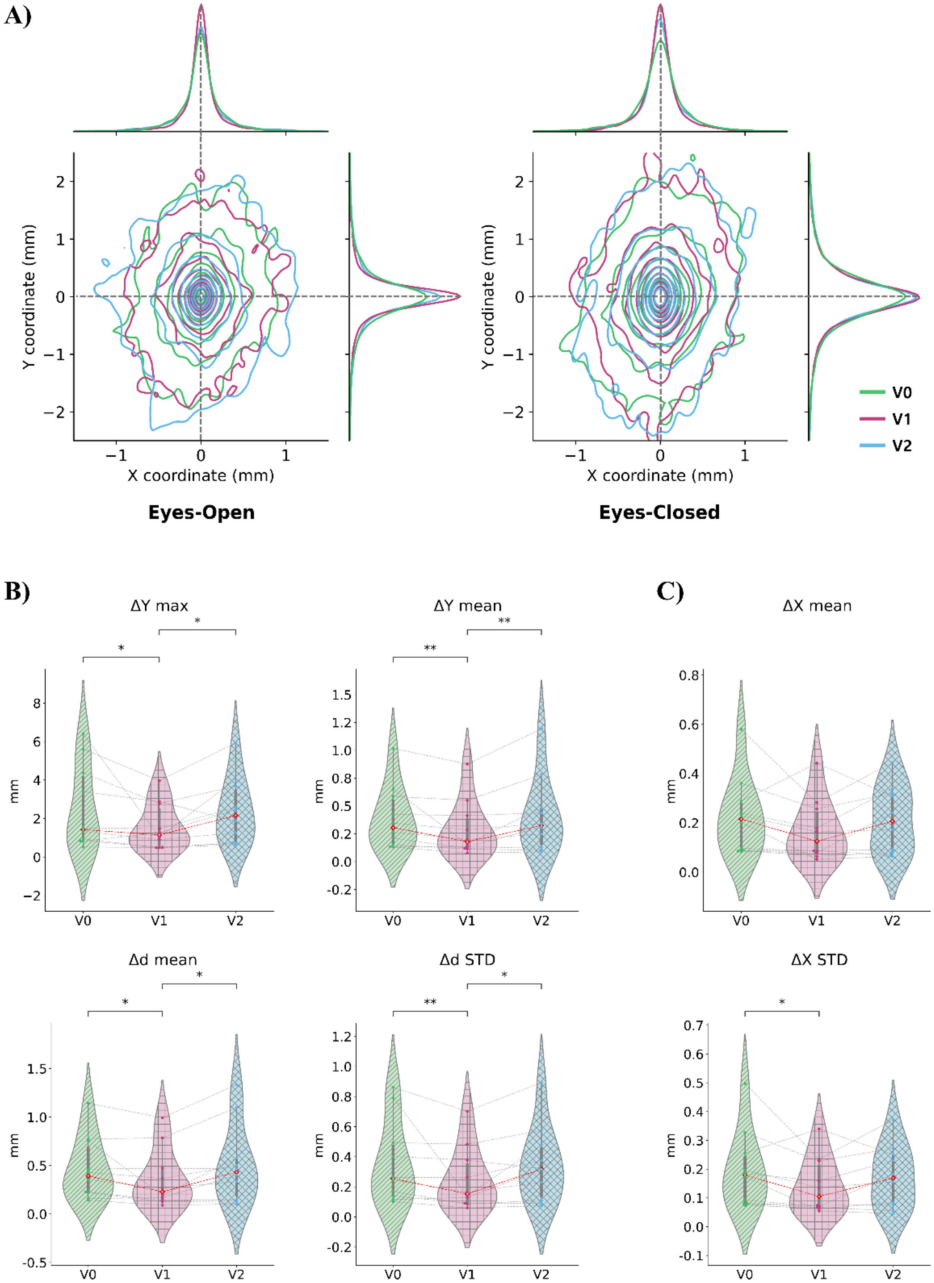
**(A)** The kernel density plots of displacement distributions for eyes-open condition (on the left side) and eyes-closed condition (on the right side). **(B,C)** The distributions of each metric across time points for eyes-open condition **(B)** and eyes-closed condition **(C)**, with post-hoc pairwise Wilcoxon signed-rank comparisons restricted to the most meaningful V0–V1 and V0–V2 contrasts (*: *p* < 0.05, **: *p* < 0.01). These plots demonstrate a marked reduction at V1 versus V0 in anterior–posterior (∆Y) and total sway (∆d) under eyes-open conditions, as well as decreased medial–lateral sway (∆X) under eyes-closed conditions. The V0–V2 comparison shows that these gains are not sustained at the 6-week follow-up, indicating transient improvement in postural stability immediately after iTBS. ∆X: medial-lateral displacement, ∆Y: anterior–posterior displacement, ∆d: total displacement (∆d), V0: baseline, V1: post treatment, V2, 6-week follow-up.

**Figure 2:**
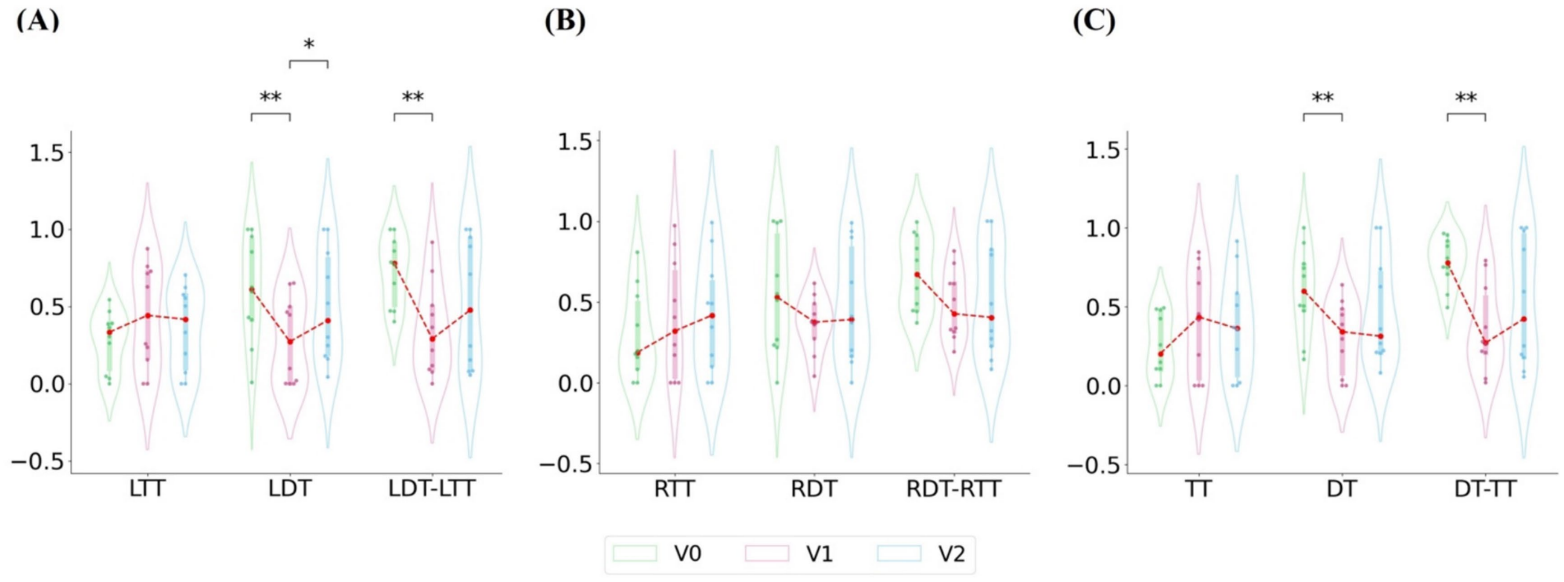
fNIRS and cognitive-motor task. **(A)** The violin plots of brain activation for right middle frontal gyrus (R. MFG) in the left toe-tapping task condition, **(B)** The violin plots of brain activation for R. MFG in the right toe-tapping task condition, **(C)** The violin plots of brain activation for R. MFG in the left/right average toe-tapping task condition, with *post-hoc* (*: *p* < 0.05, **: *p* < 0.01). **(A)** Shows the left toe-tapping task conditions (LTT / LDT /LDT–LTT); **(B)** shows the right toe-tapping task conditions (RTT / RDT /RDT–RTT); **(C)** shows the averaged condition (TT / DT/DT–TT). Violin plots depict V0 (baseline, green), V1 (post-treatment, red) and V2 (6-week follow-up, blue). Comparison bars are restricted to the most meaningful V0–V1 and V0–V2 contrasts for clarity. In the LDT condition, oxyhemoglobin (HbO) levels significantly increased at V1 compared with V0 (*p* = 0.0008), indicating enhanced prefrontal engagement. A significant increase was also observed in the dual-task difference (DT–TT) condition (*p* = 0.007), suggesting improved neural efficiency in motor–cognitive coordination. These increases returned toward baseline at V2. LTT, left toe-tapping (single-task motor condition, without mental subtraction); RTT, right toe-tapping (single-task motor condition, without mental subtraction); TT, averaged toe-tapping (mean of LTT and RTT); LDT, left dual-task (left toe-tapping with concurrent mental subtraction); RDT, right dual-task (right toe-tapping with concurrent mental subtraction); DT, averaged dual-task (mean of LDT and RDT); LDT–LTT, RDT–RTT and DT–TT denote the corresponding dual-task minus single-task difference contrasts; R.MFG, right middle frontal gyrus; HbO, oxygenated hemoglobin; V0, baseline; V1, immediately post-treatment; V2, 6-week follow-up.

However, these improvements were not maintained at V2 (Figure 1). fNIRS responses were examined separately for single-task motor conditions performed without mental subtraction (left toe-tapping, LTT; right toe-tapping, RTT; and their average, TT) and for dual-task conditions performed with concurrent mental subtraction (left dual-task, LDT; right dual-task, RDT; and their average, DT), together with the corresponding dual-task minus single-task contrasts (e.g., DT–TT). Across the single-task conditions performed without mental subtraction (LTT, RTT, TT), right middle frontal gyrus (R-MFG) oxyhemoglobin (HbO) showed no statistically significant change across time points (all *p* > 0.05; Supplementary Table S1). Figure 2 presents the fNIRS results in the R-MFG associated with task conditions, primarily focusing on within-task conditions and their contrasts. The complete fNIRS results across all task conditions, including both single- and dual-task conditions and their corresponding contrasts, are provided in Supplementary Figure S2. In contrast, the dual-task conditions performed with mental subtraction revealed transient increases in R-MFG activation at V1. Specifically, in the LDT condition, HbO levels significantly increased at V1 compared with V0 (*p* = 0.0008; see Supplementary Table S1), and the dual-task difference (DT–TT) condition also showed a significant increase at V1 (*p* = 0.007), indicating that the change in prefrontal engagement was specifically associated with the added cognitive load rather than with the motor component alone. These increases returned toward baseline levels at V2, consistent with the transient behavioral improvements observed in the BT4 metrics (Figure 2).

To explore a possible association between cortical modulation and behavioral changes, an exploratory Spearman correlation analysis was performed. A significant positive correlation was observed between the increase in oxygenated hemoglobin (Oxy-Hb) in the R-MFG during the LDT condition and the reduction in medial–lateral sway (denoted SR_ML) (SR_ML was computed as the V1-to-V0 reduction in medial–lateral sway, i.e., the V0 baseline value minus the V1 post-treatment value, such that larger positive values denote a greater reduction in sway and therefore better postural performance; see Statistical Analysis) at V1 under EC (for the mean medial–lateral sway: r = 0.733, 95% CI [0.19, 0.93], *p* = 0.016; for its standard deviation: *r* = 0.636, 95% CI [0.01, 0.90], *p* = 0.048; Supplementary Figure S3 and Supplementary Table S4). This eyes-closed medial–lateral association was pre-specified as the primary correlation, because removing visual input isolates the cerebellar–proprioceptive contribution to postural control and the medial–lateral axis is among the most consistently affected in cerebellar ataxia. Correlations between R-MFG activation and the eyes-open outcomes that also changed after stimulation (∆Y max, ∆Y mean, ∆d mean, and ∆d STD) were examined only on an exploratory basis and, given the small sample size and the number of comparisons involved, are not emphasized here; all correlations should therefore be regarded as hypothesis-generating. The full correlation results for these eyes-open outcomes are provided in Supplementary Table S5. This suggests that, in this small cohort, participants who showed a larger increase in task-related prefrontal activation also tended to show a greater reduction in postural sway. It should be emphasized, however, that the prefrontal activation was measured during a dual-task toe-tapping paradigm rather than during a postural balance task; the two measures are therefore related but distinct, and this correlation is exploratory and hypothesis-generating. It does not by itself establish a causal or compensatory mechanism, and given the small sample size it should be interpreted with caution.

## Discussion

This pilot study demonstrated that short-term, high-frequency cerebellar iTBS can transiently improve postural stability in CA patients. The selection of this iTBS protocol was based on its physiological mechanism. First, the theta-burst pattern employed in this study—triplets at 50 Hz repeated at a theta rate (5 Hz)—has been shown to induce robust and long-lasting, bidirectional modulation of cerebello-thalamo-cortical excitability (25). Notably, cerebellar TBS is known to modulate neural activity in interconnected remote cortical regions (26).

To capture these remote neuromodulatory effects, we incorporated fNIRS to directly monitor cortical activity related to cognitive-motor regulation during iTBS treatment. This approach is central to the study’s contribution: rather than relying solely on behavioral outcomes, we harnessed fNIRS as a real-time brain imaging biomarker capable of visualizing how cerebellar iTBS reshapes the cerebello-fronto-cortical loop. fNIRS provides valuable insights into cerebral hemodynamics and has been used to evaluate prefrontal engagement during balance and dual-task performance (21–23). By documenting hemodynamic changes in the prefrontal cortex, our study suggests that cerebellar stimulation may modulate not only motor output but also the broader cognitive-motor network. Our exploratory analysis revealed a positive correlation between the magnitude of task-related R-MFG activation and the degree of reduction in medial–lateral postural sway measured separately on the BT4. This association should, however, be interpreted cautiously: the prefrontal activation was recorded during a dual-task toe-tapping paradigm, which is not itself a postural-balance task, and the two measures are therefore related but not equivalent. The correlation is consistent with, but does not establish, engagement of the cerebello-fronto-cortical loop as the mechanism underlying the behavioral changes. Several non-exclusive interpretations are possible: the increased prefrontal activation may reflect a compensatory up-regulation of cortical resources, but it may equally reflect greater attentional demand, increased executive engagement, or task-related cognitive recruitment elicited by the dual-task itself, and the present design cannot disentangle these accounts. It should also be noted that the R-MFG was examined as an index of executive and attentional control rather than of limb-specific motor somatotopy. The middle frontal gyrus lies within the dorsolateral prefrontal cortex and is consistently engaged by working memory and executive demands regardless of the laterality of the motor effector (27, 28). We acknowledge, however, that executive and working-memory demands typically engage the middle frontal gyri bilaterally, and that our restriction of the primary analysis to the right MFG was a pragmatic analytic choice rather than an *a priori* hemispheric hypothesis; the implications of this regional-selection strategy are considered further in the Limitations section. Accordingly, the observation of R-MFG activation during right toe-tapping in the dual-task condition may be more parsimoniously attributed to the executive and attentional demands of the concurrent mental-subtraction component than to contralateral motor representation of the tapping foot. R-MFG activation would, tentatively, be expected under both left- and right-foot dual-task conditions and should not be read as a motor somatotopic response. Furthermore, this approach aligns with accumulating clinical evidence suggesting that cerebellar stimulation can alleviate ataxia severity. Recent meta-analyses and sham-controlled randomized studies have reported improvements in SARA scores following cerebellar stimulation, although frequency-specific results are not entirely consistent across reviews (17, 18, 29).

Notably, while significant improvements were observed in instrumental static balance parameters (BT4) and prefrontal hemodynamics (fNIRS), the K-SARA scores did not show statistically significant changes. This dissociation between clinical and instrumental outcomes underscores a key methodological insight: the early, subtle neuromodulatory changes induced by short-term iTBS are best captured not by broad clinical rating scales, but by high-precision instrument-based measures such as computerized posturography (e.g., BT4) and cortical hemodynamic biomarkers such as fNIRS. The K-SARA is a global semi-quantitative scale encompassing complex dynamic motor functions, including gait, speech, and limb coordination, domains that likely require longer-term interventions to reach the established minimal clinically important difference of 1.5 points on the SARA (30, 31). In contrast, the BT4 provides objective kinematic measurements specifically targeting static postural control, and computerized posturography of this type has been recognized as a sensitive and clinically useful tool for quantifying postural instability (32); fNIRS likewise offers a validated, portable means of directly quantifying cortical hemodynamic engagement during balance and dual-task performance (21–23). It is therefore plausible that our short-term, five-session iTBS protocol primarily modulated the neural circuits governing static postural control (as indexed by posturography rather than by the dual-task fNIRS paradigm)—a subtle physiological change captured by the high sensitivity of computerized posturography and fNIRS but insufficient to register on a global clinical scale. The convergence of these two sensitive measures revealed neurophysiological modulation that remained invisible to clinical rating alone, and integrating quantitative balance and cortical imaging assessments in future clinical trials would help identify subclinical therapeutic effects that categorical clinical scales may overlook.

Regarding the durability of effects, the improvement was observed at the V1, but were not sustained at the V2. These transient effects are consistent with previous studies in degenerative ataxia, which suggest that short-term stimulation protocols may be insufficient for lasting clinical recovery (11, 14). Given that five sessions of iTBS may not provide a sufficient intervention to consolidate neuroplastic changes, future protocols should consider extended stimulation periods and additional maintenance therapy to prolong the therapeutic benefits.

In summary, this pilot study provides preliminary evidence that short-term 50 Hz cerebellar iTBS is well tolerated and may be associated with a transient improvement in instrumented (BT4) measures of static postural stability in patients with CA, and that simultaneous fNIRS monitoring can feasibly track task-related engagement of the prefrontal cortex during cognitive-motor performance. The convergent changes in instrumented balance metrics and prefrontal hemodynamics are consistent with modulation of the cerebello-fronto-cortical loop, but—given the dual-task (rather than balance-specific) nature of the fNIRS paradigm—they should be regarded as hypothesis-generating rather than as direct visualization of the underlying mechanism. The cerebellar iTBS protocol was well-tolerated with no serious adverse events, supporting its safety and feasibility in this population. However, given the single-arm design without sham control and susceptibility of performance-based balance testing to practice and expectancy effects, findings should be interpreted as preliminary and do not allow causal inferences. These results warrant evaluation of high-frequency cerebellar iTBS as a potential adjunct to rehabilitation in randomized, sham-controlled trials, with fNIRS as a pre-specified neurophysiological outcome measure to elucidate stimulation mechanisms, and with longitudinal follow-up. In alignment with the Research Topic’s emphasis on integrating brain imaging with neuromodulation to elucidate cortical mechanisms of motor control, this proof-of-concept study suggests that fNIRS may serve as a feasible, real-time imaging biomarker for monitoring iTBS-induced changes in the cerebello-fronto-cortical network, paving the way for its incorporation as a standard neurophysiological outcome measure in future multimodal neuromodulation trials targeting movement disorders.

## Limitations

It is essential to contextualize this study within its intended scope: this investigation was designed as a Phase 1, proof-of-concept (PoC) study, not a definitive clinical trial. Its primary objectives were to (1) confirm the safety and tolerability of 50 Hz cerebellar iTBS in patients with cerebellar ataxia, and (2) establish the feasibility and preliminary validity of an fNIRS-based assessment protocol for capturing cortical biomarkers of iTBS-induced neuromodulation. This PoC phase is a scientifically necessary prerequisite before committing resources to a large-scale randomized controlled trial (RCT). Within this intended scope, several limitations should be explicitly acknowledged. First, the absence of a sham-controlled condition substantially limits causal inference. Because performance-based balance measures are vulnerable to learning, adaptation, and placebo effects, iTBS-specific effects cannot be fully disentangled from nonspecific changes without a controlled comparison. Although the convergent findings across the objective kinematic BT4 data and the fNIRS cortical hemodynamics provide mutually reinforcing evidence of a neurophysiological signal, they do not substitute for a sham-controlled design; the present results must therefore be interpreted as preliminary and hypothesis-generating rather than as definitive evidence of efficacy. Second, the heterogeneous composition of the cohort (MSA-C, *n* = 5; SAOA, *n* = 4; SCA6, *n* = 1) introduces variability in baseline postural instability and potential treatment response. MSA-C patients in particular may have limited plastic potential due to multi-system neurodegeneration, and their parkinsonian features (e.g., rigidity, bradykinesia, postural instability) may independently affect balance outcomes, potentially modulating the therapeutic response to iTBS. The potential for differential therapeutic responses across these subgroups should therefore be considered when interpreting the present findings. Third, the small sample size (*n* = 10) limits statistical power and generalizability, increasing the risk of Type II errors, particularly for clinical scales such as the K-SARA. Fourth, the prefrontal region of interest in the primary analysis was restricted to the right middle frontal gyrus; because executive and working-memory demands typically engage the middle frontal gyri bilaterally, this right-lateralized selection may have under-represented bilateral prefrontal contributions, and future analyses should examine bilateral regions of interest. Although the total pulse number exceeded conventional parameters, all sessions strictly adhered to the updated TMS safety framework (24) and were approved by the institutional IRB, confirming the safety profile required to proceed to a controlled trial. Based on the safety and feasibility data obtained here, future work will employ randomized, sham-controlled, double-blinded designs with pre-specified primary endpoints, incorporating both fNIRS and kinematic measures as co-primary neurophysiological outcomes to rigorously test the mechanistic hypothesis generated by this PoC study.

## Data Availability

The raw data supporting the conclusions of this article will be made available by the authors, without undue reservation.
