# Supplementary material for: Feasibility and preliminary signal of cerebellar intermittent theta burst stimulation for static balance in cerebellar ataxia: a pilot study
Source: Front Neurol. 2026 Jul 15;17:1826602. doi: 10.3389/fneur.2026.1826602 (PMC13414872; doi:10.3389/fneur.2026.1826602)
Supplement: Supplementary file 1 [file data_sheet_1.docx]

**Supplementary Information**

**Data Acquisition** (Fig S1) Four types of data were collected at three time points: baseline (V0), after five consecutive rTMS sessions (V1), and at a 6-week follow-up (V2).

**Clinical Assessment**

Ataxia severity, activities of daily living (ADL), and quality of life were evaluated using the Korean versions of the Scale for the Assessment and Rating of Ataxia (K-SARA), Modified Barthel Index (K-MBI), and 36-Item Short Form Health Survey (SF-36-K), respectively.

K-SARA: Evaluates gait, stance, sitting, speech, finger chase, nose-finger test, alternating hand movements, and heel-shin slide. Higher scores indicate greater motor impairment.

K-MBI: Includes 11 ADL items (e.g., hygiene, dressing, feeding, mobility, and bladder/bowel control). Higher scores indicate greater independence.

SF-36-K: Comprises physical and mental health domains, each with four subscales. Higher scores reflect better perceived health status.

**Balance Assessment**

Static balance control was measured for 30 seconds under both eyes-closed (EC) and eyes-open (EO) conditions using a Balance Trainer 4 (BT4; HUR, Finland). The anterior-posterior (Y-axis) and medial-lateral (X-axis) displacement of the center of pressure (COP) were sampled at 10 ms intervals. The primary metrics derived from the BT4 included the Romberg quotient (RQ), trace length of swaying, area of the 90% confidence ellipse (C90), angle of the C90, velocity, and standard deviation (STD) of velocity, as well as the maximum, minimum, mean, and STD of X- and Y-axis sway displacement. All BT4-derived features—including velocity, total displacement (Δd), and the Romberg quotient—were entered into the Friedman analysis reported in the main text (Table 2). Of these, only ΔY (anterior–posterior), ΔX (medial–lateral), and Δd reached statistical significance at V1 in the relevant viewing conditions; velocity and RQ did not show significant changes across time points and were therefore not discussed further in the main text. Weight distribution was also assessed for both EC and EO conditions. The Romberg quotient quantifies balance degradation when visual input is removed.

**fNIRS task**

The NIRSIT Lite device (OBELAB, South Korea) measures changes in oxy- (HbO₂) and deoxy hemoglobin (HbR) concentrations using 15 prefrontal channels (5 sources, 13 detectors). Data were acquired at rest for 300 s (fixation cross on a black background) to establish a baseline. Each participant completed two motor task conditions to allow direct comparison of pure motor processing with motor–cognitive integration. In the single-task (motor-only) condition, participants performed continuous left or right toe-tapping for 60 s without any concurrent cognitive load. In the dual-task condition, participants performed the same toe-tapping while simultaneously performing continuous mental subtraction by repeatedly subtracting three from a randomly given number (e.g., "1000 – 3 – 3 – 3 …"). After each response, the experimenter verbally presented a new two-digit number for subtraction. This single-task / dual-task design allowed the cognitive cost of the additional executive load to be isolated by contrasting the two conditions (dual-task minus single-task), thereby providing a sensitive index of motor–cognitive integration and sensorimotor recruitment.

**Data Analysis**

- **BT4 Data Preprocessing**: Initial COP coordinates were normalized to the origin to minimize positional variability. Displacement features (∆X, ∆Y, ∆d) and 90% ellipse (C90) parameters were recalculated. Derived features included maximum, minimum, mean, and standard deviation of each axis, as well as velocity-based metrics.
- **fNIRS Preprocessing**: Signals were processed using NIRSIT Quest Tool (v1.0.5). Only HbO₂ signals were used, because they directly reflect the brain activity. Preprocessing included invalid channel rejection, low-intensity high-variation channel rejection, motion artifact correction, and sixth-order bandpass filtering (0.005 – 0.1 Hz). (Fishburn et al., 2019) Signals were converted to NIFTI format using 3D-diffuse optical tomography (DOT) and analyzed with a general linear model (SPM12). To assess cortical activation patterns during motor-cognitive interaction, beta values were extracted from 14 prefrontal regions defined by the Brainnetome atlas using MarsBar toolbox.
- **Statistical Analysis**: The Mann-Whitney U test was used to investigate the influence of sex on rTMS efficacy over time for all measured features. Hedge’s adjusted g was calculated as the effect size, which provides a more reliable estimate than Cohen’s d for small sample sizes. Partial correlation analysis was then performed to assess the effects of: (1) age, with sex and disease duration as covariates; (2) disease duration, with sex and age as covariates; and (3) ataxia severity (measured by clinical scores), with sex, age, and disease duration as covariates. The K-MBI was excluded from this analysis due to a lack of variation across time points for most patients. To evaluate changes over time, the Friedman test (a non-parametric repeated measures test) was employed, with Kendall’s coefficient of concordance used to determine the effect size. Post-hoc analysis was conducted using the Wilcoxon signed-rank test. All statistical analyses were performed using Python (version 3.11.9).

In contrast, the single-task motor conditions performed without concurrent mental subtraction (LTT, RTT and their average TT) showed no significant changes across time points (all p > 0.05), confirming that pure motor execution did not by itself produce a measurable change in prefrontal activation following iTBS. Although a mild upward trend was noted in the right dual-task (RDT) condition (p = 0.301), it did not reach statistical significance. Together, these results indicate that the iTBS-related increase in prefrontal engagement was specific to the dual-task (motor + cognitive) condition rather than to motor execution alone. Abbreviations: ΔX, medial-lateral displacement; ΔY, anterior-posterior displacement; Δd, total displacement; V0, baseline; V1, post-treatment; V2, 6-week follow-up; HbO, oxygenated hemoglobin; R.MFG, right middle frontal gyrus.

**fNIRS Results**


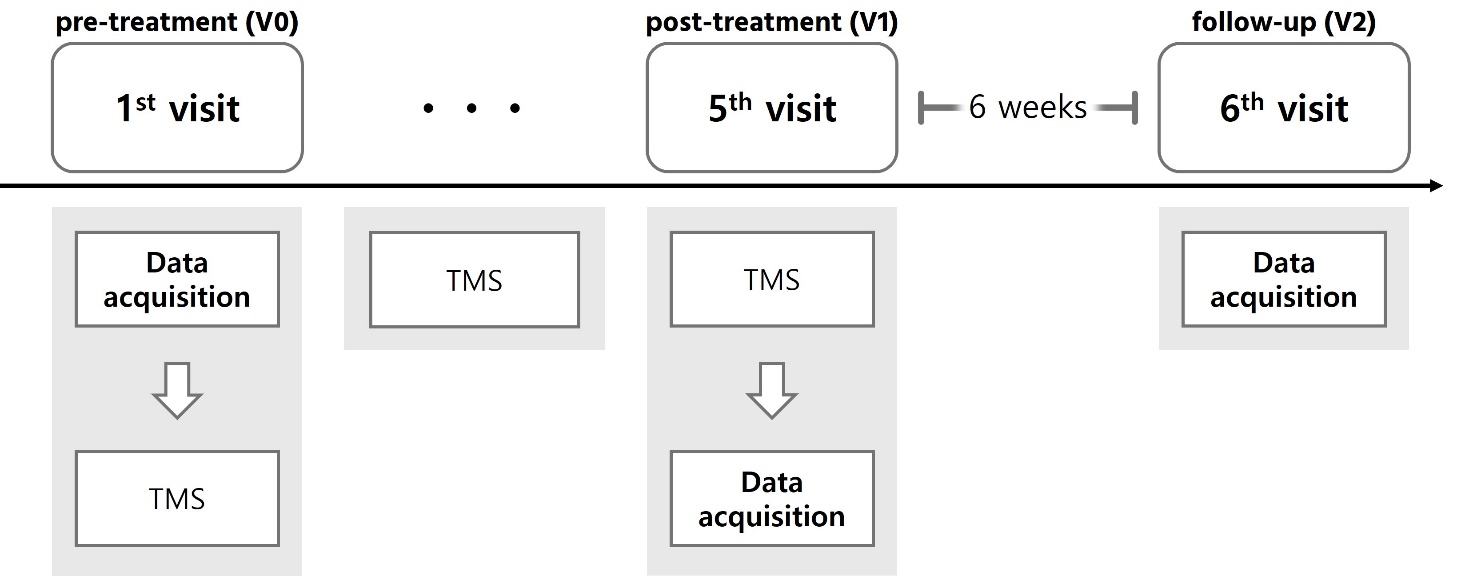


Figure S1 Experiment Protocol

###
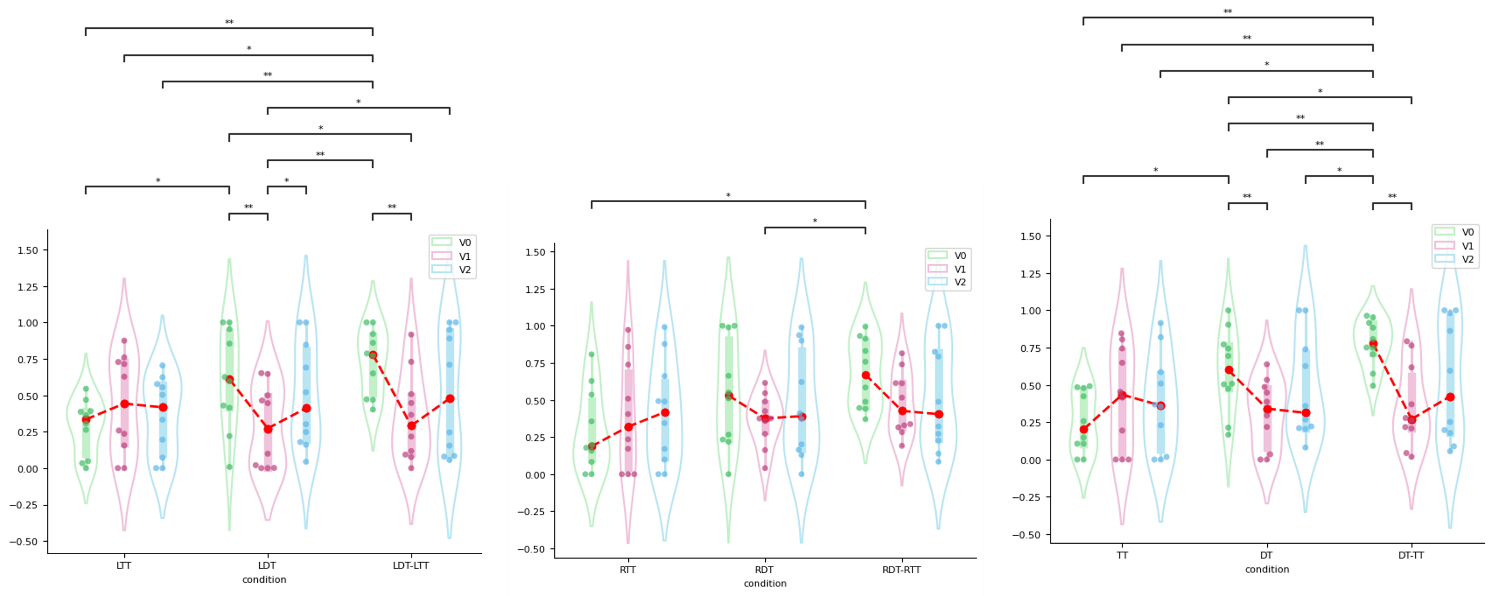


**Figure S2. fNIRS and cognitive-motor task**

**Left) Violin plots of right middle frontal gyrus (R.MFG) HbO activation in the left toe-tapping task condition (LTT = single-task motor only; LDT = left toe-tapping with concurrent mental subtraction; LDT–LTT = dual-task minus single-task contrast). Center) The same for the right toe-tapping task condition (RTT = single-task motor only; RDT = right toe-tapping with concurrent mental subtraction; RDT–RTT = dual-task minus single-task contrast). Right) The averaged condition (TT = mean of LTT and RTT; DT = mean of LDT and RDT; DT–TT = dual-task minus single-task contrast). Post-hoc Wilcoxon signed-rank tests: *: p<.05, **: p<.01. In the left dual-task condition (LDT), HbO levels significantly increased at post-intervention (V1) compared to baseline (V0) (p = 0.0008), indicating enhanced prefrontal engagement specifically associated with the added cognitive demand. A significant increase was also observed in the dual-task minus single-task contrast (DT–TT) (p = 0.007), demonstrating that the change in prefrontal activation is attributable to the cognitive component rather than to the motor component alone.**

**
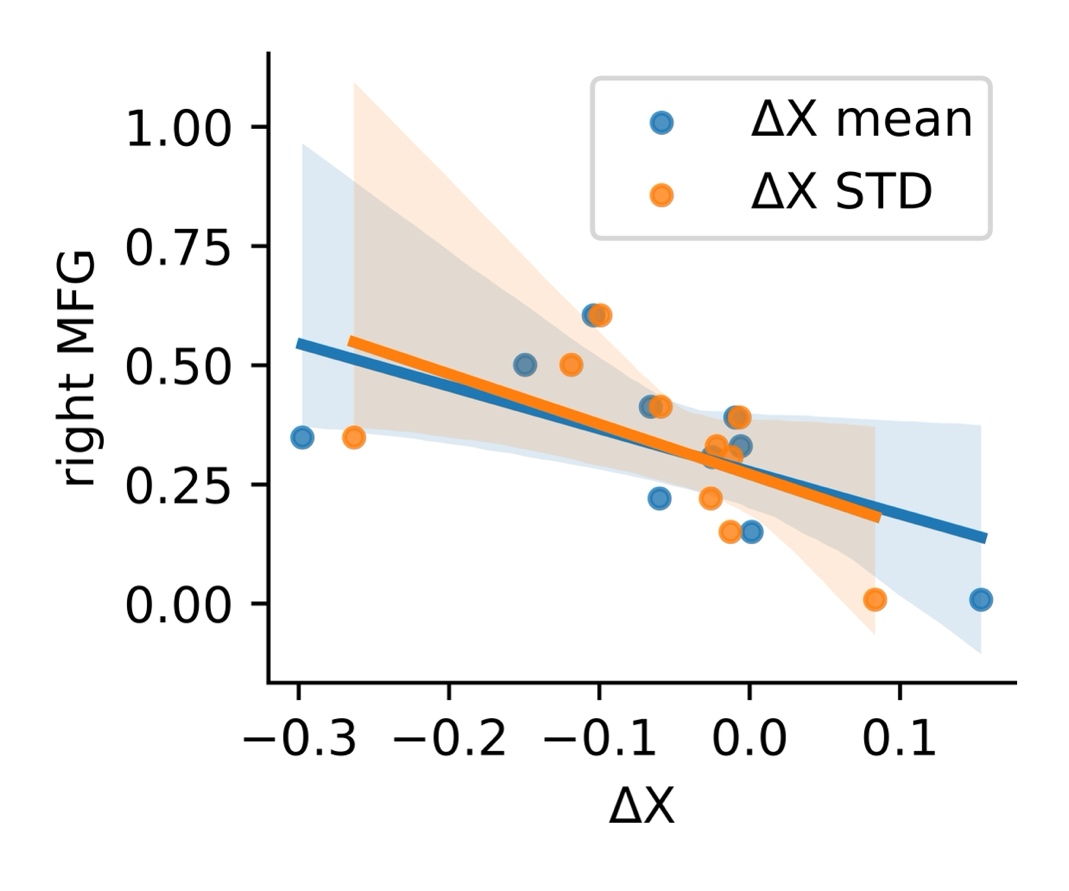
**

**Figure S3. Correlation between rMFG activation and ∆X**

**Scatter plot showing the correlation between right MFG activation changes during the LDT condition and the V1−V0 change in medial–lateral sway (ΔX (V1−V0)) during the EC condition. The mean ΔX (V1−V0) (blue; r=−0.733, p=0.016) and the STD ΔX (V1−V0) (orange; r=−0.636, p=0.048) showed a negative correlation. For detail, see Table S4. Here, the plotted variable is the V1−V0 change in medial–lateral sway (ΔX (V1−V0); the post-treatment minus baseline value, V1 − V0), so that a negative correlation indicates that a larger increase in R-MFG activation was associated with a greater reduction in sway, i.e., better postural performance. This ΔX (V1−V0) change variable is distinct from the per-visit ∆X displacement reported in Supplementary Table S2.**

**
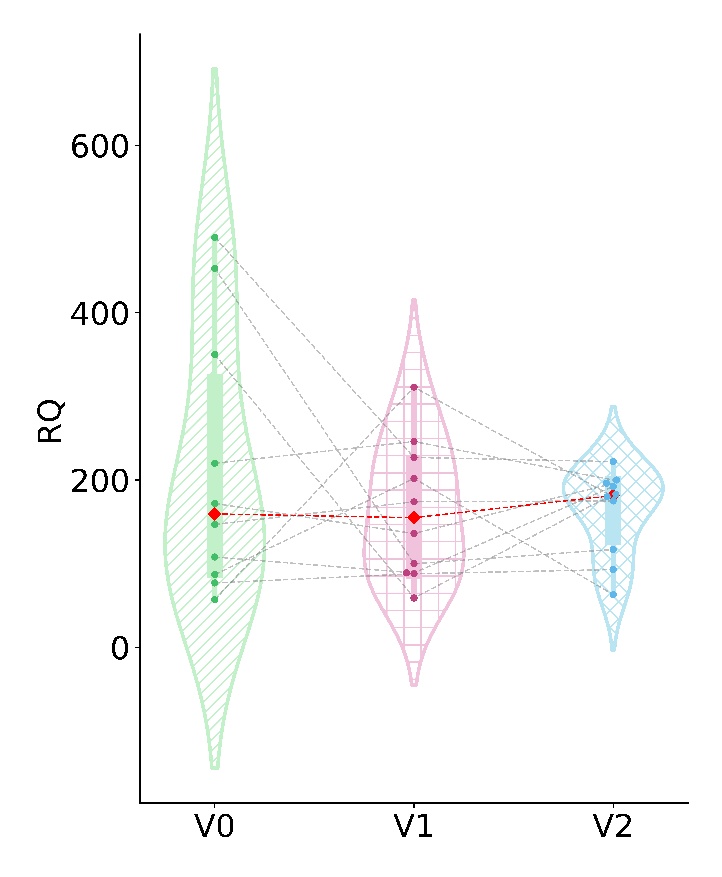
**

**Figure S4. Distribution of Romberg quotient (RQ) at V0, V1 and V2**

**Violin plots showing the RQ at each time point. The change across the three time points was statistically significant based on the Friedman test (*p*=.0004). However, no significant differences are shown in the post-hoc analysis.**

### Supplementary **Table S1. Friedman statistics for right middle frontal gyrus (R.MFG) oxyhemoglobin (HbO) activation in each toe-tapping condition (V0/V1/V2). This table is complementary to, and does not overlap with, main-text Table 1 (which reports demographic and clinical scale data). LTT and RTT denote single-task motor conditions (left and right toe-tapping without mental subtraction); LDT and RDT denote the corresponding dual-task conditions with concurrent mental subtraction; LDT–LTT, RDT–RTT, and DT–TT denote the dual-task minus single-task contrasts that isolate the cortical cost of the added cognitive load.**

|  | **W** | **Q** | **p-unc.** |
| --- | --- | --- | --- |
| LTT | 0.03 | 0.6 | 0.741 |
| LDT | 0.715 | 14.308 | **0.0008** |
| LDT-LTT | 0.57 | 11.4 | **0.003** |
| RTT | 0.01 | 0.2 | 0.905 |
| RDT | 0.07 | 1.4 | 0.497 |
| RDT-RTT | 0.12 | 2.4 | 0.301 |
| TT | 0.018 | 0.359 | 0.836 |
| DT | 0.57 | 11.4 | **0.003** |
| DT-TT | 0.49 | 9.8 | **0.007** |

Supplementary Table S2. Balance Trainer 4-derived features at V0, V1 and V2 in both EO and EC conditions (relocated from the former main-text Table 2 to reduce redundancy with Figure 1). Values are expressed as mean ± SD. Friedman Q statistics represent the overall three-time-point comparison; post-hoc Wilcoxon signed-rank pairwise p-values for V0–V1 and V0–V2 are reported in the main-text Results. (*: p<.05).

|  | | V0 (baseline) | | V1 (post-treatment) | | V2 (6-week follow-up) | | Difference (Q) | |
| --- | --- | --- | --- | --- | --- | --- | --- | --- | --- |
|  |  | **EO** | **EC** | **EO** | **EC** | **EO** | **EC** | **EO** | **EC** |
| Trace Length (mm) | | 1399.511 ±981.625 | 1615.889 ±1041.000 | 1082.104 ±918.161 | 1442.941 ±1027.535 | 1447.847 ±1277.908 | 1600.076 ±1163.055 | 2.6 | 0.6 |
| Velocity (mm/ms) | | 46.649 ±32.723 | 53.864 ±34.670 | 36.072 ±30.606 | 48.098 ±34.252 | 48.262 ±42.598 | 53.336 ±38.769 | 2.6 | 0.6 |
| ∆ X  (mm) | Max | 1.021 ±0.822 | 1.232 ±0.912 | 0.798 ±0.736 | 0.868 ±0.543 | 1.017 ±0.928 | 1.005 ±0.761 | 1.4 | 5.4 |
|  | Mean | 0.181 ±0.130 | 0.226 ±0.158 | 0.149 ±0.132 | 0.170 ±0.125 | 0.196 ±0.194 | 0.189 ±0.152 | 1.4 | **6.2*** |
|  | STD | 0.153 ±0.114 | 0.196 ±0.135 | 0.128 ±0.114 | 0.143 ±0.097 | 0.984 ±2.564 | 1.259 ±3.442 | 1.4 | **7.2*** |
| ∆ Y  (mm) | Max | 2.578 ±2.198 | 2.729 ±2.058 | 1.578 ±1.212 | 2.098 ±1.564 | 2.200 ±1.833 | 2.547 ±1.628 | **8.6*** | 2.4 |
|  | Mean | 0.392 ±0.287 | 0.443 ±0.286 | 0.293 ±0.255 | 0.412 ±0.308 | 0.381 ±0.369 | 0.441 ±0.358 | **8.6*** | 1.4 |
|  | STD | 0.361 ±0.280 | 0.395 ±0.264 | 0.255 ±0.214 | 0.357 ±0.262 | 0.336 ±0.273 | 0.411 ±0.278 | 5.6 | 0.2 |
| ∆ d  (mm) | Max | 2.631 ±2.190 | 2.807 ±2.073 | 1.623 ±1.265 | 2.159 ±1.595 | 2.260 ±1.827 | 2.586 ±1.605 | **8.6*** | 2.4 |
|  | Mean | 0.467 ±0.327 | 0.539 ±0.347 | 0.361 ±0.306 | 0.481 ±0.343 | 0.483 ±0.426 | 0.533 ±0.388 | 2.6 | 0.6 |
|  | STD | 0.359 ±0.277 | 0.396 ±0.261 | 0.250 ±0.210 | 0.348 ±0.253 | 0.326 ±0.265 | 0.394 ±0.275 | **7.2*** | 0.6 |

∆X: medial-lateral displacement, ∆Y: anterior-posterior displacement, ∆d: total displacement, EC: eyes-closed condition, EO: eyes-open condition, Q: the Friedman chi-square statistic, STD: standard deviation

Supplementary Table S3. Romberg quotient (RQ) at V0, V1 and V2, with Friedman three-time-point statistics. RQ was derived from the BT4 recordings and showed a statistically significant change across time points. However, post-hoc did not show any significant difference.

|  | V0 | V1 | V2 | Friedman Q | p |
| --- | --- | --- | --- | --- | --- |
| RQ | 216.1 ± 159.451 | 163.2 ± 82.425 | 154.2 ± 53.243 | 18.12 | 0.0004 |

Supplementary **Table S4. Spearman Correlation between rMFG activation and ∆X**

|  | **n** | **r** | **CI 95%** | **p-val** | **Cohen’s d** |
| --- | --- | --- | --- | --- | --- |
| **Mean** | 10 | −0.733 | [−0.93 −0.19] | 0.016 | -1.851 |
| **STD** | 10 | −0.636 | [−0.90 −0.01] | 0.048 | -1.991 |

Supplementary Table S5. Exploratory Spearman correlations between the V1-to-V0 increase in R-MFG oxygenated haemoglobin (LDT condition) and the V1-to-V0 change in the eyes-open balance outcomes that changed after stimulation (∆Y max, ∆Y mean, ∆d mean, ∆d STD). All correlations were non-significant (all p > 0.05).

| Eyes-open outcome (V1-to-V0 change) | n | r | 95% CI | p |
| --- | --- | --- | --- | --- |
| ∆Y max | 10 | -0.370 | [ -0.81 0.34] | 0.293 |
| ∆Y mean | 10 | -0.103 | [-0.69 0.56] | 0.777 |
| ∆d mean | 10 | 0.212 | [-0.48 0.74] | 0.556 |
| ∆d STD | 10 | -0.2 | [-0.74 0.49] | 0.580 |

### **References**

Schmitz-Hübsch T, du Montcel ST, Baliko L, Berciano J, Boesch S, Depondt C, et al. Scale for the assessment and rating of ataxia: development of a new clinical scale. Neurology. (2006) 66:1717–20. doi: 10.1212/01.wnl.0000219042.60538.92

Jung HY, et al. Development of the Korean version of Modified Barthel Index (K-MBI): multi-center study for subjects with stroke. J Korean Acad Rehabil Med. (2007) 31:283–97.

Ware JE Jr, Sherbourne CD. The MOS 36-item short-form health survey (SF-36): I. Conceptual framework and item selection. Med Care. (1992) 30:473–83. doi: 10.1097/00005650-199206000-00002

Kim SH, Jo MW, Lee S. Psychometric properties of the Korean SF-36 v2. Asian Nurs Res. (2013) 7:61–6.

Fishburn FA, Ludlum RS, Vaidya CJ, Medvedev AV. Temporal derivative distribution repair (TDDR): a motion correction method for fNIRS. NeuroImage. (2019) 184:171–9. doi: 10.1016/j.neuroimage.2018.09.025
